# Does shared decision making increase prostate screening uptake in countries with a low prevalence of prostate cancer?

**DOI:** 10.4314/ahs.v20i4.43

**Published:** 2020-12

**Authors:** Hussein Saad Amin, Mostafafa Ahmed Arafa, Karim Hamda Farhat, Danny Munther Rabah, Abdulaziz Abdullah Altaweel, Abdulaziz Hamed Alhammad

**Affiliations:** 1 Department of Family and Community Medicine, College of Medicine, King Saud University, Riyadh, Saudi Arabia; 2 Cancer Research Chair, College of Medicine, King Saud University, Riyadh, Saudi Arabia; 3 Surgery Department, College of Medicine, King Saud University, Riyadh, Saudi Arabia; 4 Medical Intern, College of Medicine, King Saud University, Riyadh, Saudi Arabia

**Keywords:** Decision making, prostate cancer screening, low prevalence countries

## Abstract

**Background:**

Men over 50 should discuss the benefits and harms of prostate-specific antigen (PSA) testing with their doctors.

**Objectives:**

To investigate whether shared decision making (SDM) increases the uptake of prostate cancer screening practices among Saudi men.

**Methods:**

This community-based study recruited men aged ≥ 50 years between January and April 2019. Sociodemographic characteristics, history, and current medical condition information were collected. SDM information with regards to prostate cancer screening was discussed.

**Results:**

In total, 2034 Saudi men, aged between 50 and 88 years, agreed to participate in the current study. Prostate examination for early detection of cancer was recommended for 35.4% (720) of subjects. Of the subjects, 23.3% (473) reported that the physicians discussed the advantages and benefits of PSA testing, whereas only 5.6% (114) stated that the physicians explained the disadvantages and drawbacks of PSA testing.

**Conclusion:**

Our findings suggest that less than one fourth discussed the advantages and disadvantages of PSA testing with their physicians; of these, less than one third underwent PSA blood tests. Improvements are needed in SDM for and against PSA screening. SDM does not affect the intensity of PSA testing. Primary health care physicians should be actively involved in the SDM process.

## Background

Prostate cancer is one of the major issues related to men's health worldwide. The World Health Organization (WHO) has reported that prostate cancer is the second most commonly diagnosed type of cancer in men and the fifth leading cause of death in men worldwide^1^. It accounts for 6.6% of the total mortality in men, and by 2030, prostate cancer is estimated to cause 1.7 million cases and 499,000 new deaths globally 2.

The incidence of prostate cancer is significantly lower in Saudi Arabia and the Gulf region than in the USA and European countries 3; however, its incidence has been increasing. In addition, many metastatic cases have been recently diagnosed in patients aged <50 years 4.

Over diagnosis and overtreatment of prostate cancer are major concerns at the population level, but it is difficult to ascertain who has been over diagnosed or over treated at an individual level. Policies are needed to decrease over diagnosis and/or to uncouple over diagnosis from overtreatment 5.

The benefits and harms of prostate cancer screening have been debatable. Prostate cancer screening does not “save lives” in terms of reducing overall mortality, but it may reduce the risk of prostate cancer mortality. Based on the new US Preventive Services Task Force (USPSTF) guidelines, men aged 50 years and older should discuss the benefits and harms of prostate-specific antigen (PSA) testing with their doctors 6. This process is called shared decision making (SDM).

## Definition of SDM

“It is the process of interacting with patients who wish to be involved in arriving at an informed, values-based choice among two or more medically reasonable alternatives.” Here, “informed” refers to the fact that there is choices, options, and information about benefits and harms of the options are available to the patients. “Value-based” is defined as “what is important to the patient”^7^.

In clinical practice, SDM is often encouraged as the essential constituent of all patient-provider connections with regards to medical and health choices 7, 8; this is because SDM is based on values of patient-centered care 9, 10. SDM is particularly recommended for “preference-sensitive medical decisions” 11 and considered essential for screening and treatment of prostate cancer^12^. Most professional organizations, including the American Cancer Society (ACS), American Urological Association (AUA), American College of Physicians (ACP), and U.S. Preventive Services Task Force (USPSTF), accentuate that PSA testing should not occur before a thorough discussion between the health-care provider and patient about the known risks and potential benefits of the test. Guidelines strongly advise health-care providers to involve patients, principally those at elevated risk of prostate cancer, in a “shared decision making” process about PSA testing 13.

Studies that document the kinds of conversations men are having with their health-care providers about PSA testing are scarce. Understanding the extent to which men are engaged in SDM is important particularly in our region, in order to help progress toward the healthy men. This study aimed to assess the implementation of SDM, to investigate whether it increased the uptake of prostate cancer screening practices among Saudi men,

## Methods

This community-based study invited the participation of men aged ≥50 years. They were recruited from primary health care outpatient clinics at different hospitals, big malls, and through a google survey. Men aged <50 years and those with prostate cancer history were excluded from the study. The purpose and rationale of the study were explained to the subjects in detail, and their written informed consent was obtained. They were subjected to a questionnaire that collected data about sociodemographic characteristics; medical history; history of prostate cancer screening, PSA testing, and prostate examination; and present medical condition. SDM information with regards to prostate cancer screening, was discussed in detail with them.

## Statistical analysis

SPSS version 20 was used for analysis. Simple frequency distribution was used to analyze different variables. The chi-square test and unpaired t test were employed to determine the association between qualitative and quantitative data. The significance level was set at 0.05%.

## Results

In total, 2034 Saudi men agreed to participate in the current study. Age of the subjects ranged from 50 to 88 years, with a mean age of 57.9±8.6 years. Of the subjects, 89.9% had a secondary or university degree. Of the subjects, 25.1% mentioned that they were suffering from prostate-related problems; 95.8% had benign prostate hyperplasia and prostatitis. Only 4.2% of subjects mentioned that they had prostate cancer.

Table 1 illustrates the responses of the subjects with regards to SDM statements. Of the subjects, 35.4% (720) were advised to undergo prostate examination at the age of 50 years for early detection of prostate cancer. Of the subjects, 23.3% (473) mentioned that the physicians discussed the advantages and benefits of PSA testing, whereas only 5.6% (114) stated that the physicians discussed the disadvantages and drawbacks of PSA testing. Among the subjects who were advised to undergo PSA testing and prostate examination, 28.9% (208) underwent the tests whereas 7.9% 14 underwent the tests annually. Patients who were advised about the benefits of early prostate cancer examination were significantly younger (mean age, 56.6±8.3 years) and had a higher degree of education than those who were not involved in informed decision making (mean age, 60.4±8.6 years) (t=5.59, p=0.00). Of the subjects, 52.3% of participants were advised to undergo prostate cancer examination and PSA testing by the urologist, whereas 26.7% of subjects mentioned that the primary health care physicians had discussed the benefits and harms of PSA testing with them.

**Table 1:** Participants' responses regarding shared decision making for early cancer prostate screening

Statements	Yes	%	No	%
Has your doctor advised you to undergo early prostate cancer examination?	720	(35.4%)	1314	(64.6%)
Have you discussed the benefits and advantages of PSA testing with your physician?	473	(23.3%)	1561	(76.7%)
Have you discussed the drawbacks of PSA testing with your physician?	114	(5.6%)	1920	(94.4%)

## Discussion

The current study findings add to an increasing indication that SDM is an unusual occurrence in PSA screening. Mostly, PSA screening occurs with partial or no discussion about the associated advantages and disadvantages between the physician and patient. The majority of participants in the current study were not advised about the importance and significance of early prostate cancer examination (at the age of 50 years), were not recommended to undergo PSA testing, and were not provided information regarding the advantages and drawbacks of PSA blood testing.

Ongoing debates have shown that the aim of SDM has not yet been clarified. Some view SDM as a partnership between patient and/or patient care-related parties and physicians or healthcare providers in terms of equally sharing healthcare-related decisions 15, 16. For others, SDM is a procedure that is involved in decision making^17^ or a tactic to integrate preference-sensitive elements in decision making 14.

The USPSTF recommends that men should have an opportunity to discuss the potential benefits and harms of prostate cancer screening with their clinician and to incorporate their values and preferences in this decision^18^,^19^.

Different studies on this topic have shown varied results. Fedewa at al. discussed the recent patterns in SDM for PSA testing in the United States and concluded that 58.5% and 62.6% of subjects reported that they received ≥1 element of SDM in 2010 and 2015, respectively. However, a shift from only being told about the advantages of PSA testing toward full SDM was observed^20^. Pucheril et al. concluded that, despite the recommendation that physicians should engage patients in the SDM process, less than a third of the subjects were advised about the advantages and disadvantages of PSA testing 21. A national study in the USA found that most men reported slight SDM during PSA screening and found that the deficiency in SDM was more prevalent in non-screened men than in screened men 22. Of the Saudi men who received consultations regarding early prostate cancer examination, only 28.9% underwent PSA testing, of which 7.9% underwent the test annually (if necessary). One study reported that, among men who underwent PSA testing recently, 58.5% and 62.6% reported that they received ≥1 element of SDM in 2010 and 2015, respectively 20. A combined analysis of two practice-based randomized controlled trials showed that SDM interventions can increase men's knowledge, alter their perceptions of prostate cancer screening, and reduce actual screening. However, these interventions may not guarantee an increase in shared decisions 23.

Primary healthcare physicians did not have a major role in SDM consultation, because only 26.6% of the respondents mentioned that they discussed the benefits and harms of PSA testing with their physicians. This value could be representative of the physicians' knowledge and attitude toward counselling. It was also found that physicians, particularly primary health care physicians, who were influenced by scientific evidence were likely to practice informed decision making with their patients 24. This variability in the physicians' use of informed decision making processes can be attributed to their beliefs and attitudes about screening, which in turn could affect their practice techniques and counseling^25^, ^26^. Although most recommendations encourage SDM for prostate cancer screening, finding the time for these discussions in a full practice is difficult.

## Limitations

Firstly, although the subjects represented the Saudi population, the sample size was not big enough; ideally, the study population should have included Saudi men from different regions. Secondly, the timings of consultation and PSA testing were not evaluated; thus, we could not determine whether the consultations were directly responsible for PSA testing. Finally, the issue of late or delayed cancer diagnosis and its relation to SDM was not addressed in this study.

## Conclusion

Our findings suggest that less than one fourth of subjects discussed the advantages and disadvantages of PSA testing with their physicians; of these, less than one third underwent PSA blood tests. Improvements are needed in SDM for and against PSA screening. SDM does not affect the intensity of PSA testing. Primary health care physicians should be actively involved in the SDM process as they are the direct points of contact with the healthcare system for an individual. Future researches should be directed qualitative studies that strengthen the discussions related to prostate cancer screening between patients and their physicians.
